# On the origin of the histone fold

**DOI:** 10.1186/1472-6807-7-17

**Published:** 2007-03-28

**Authors:** Vikram Alva, Moritz Ammelburg, Johannes Söding, Andrei N Lupas

**Affiliations:** 1 Department of Protein Evolution, Max-Planck-Institute for Developmental Biology, Spemannstr. 35, D-72076 Tübingen, Germany

## Abstract

**Background:**

Histones organize the genomic DNA of eukaryotes into chromatin. The four core histone subunits consist of two consecutive helix-strand-helix motifs and are interleaved into heterodimers with a unique fold. We have searched for the evolutionary origin of this fold using sequence and structure comparisons, based on the hypothesis that folded proteins evolved by combination of an ancestral set of peptides, the antecedent domain segments.

**Results:**

Our results suggest that an antecedent domain segment, corresponding to one helix-strand-helix motif, gave rise divergently to the N-terminal substrate recognition domain of Clp/Hsp100 proteins and to the helical part of the extended ATPase domain found in AAA+ proteins. The histone fold arose subsequently from the latter through a 3D domain-swapping event. To our knowledge, this is the first example of a genetically fixed 3D domain swap that led to the emergence of a protein family with novel properties, establishing domain swapping as a mechanism for protein evolution.

**Conclusion:**

The helix-strand-helix motif common to these three folds provides support for our theory of an 'ancient peptide world' by demonstrating how an ancestral fragment can give rise to 3 different folds.

## Background

The organization of DNA into chromatin allows its compact and reversible packaging into the nucleus of a eukaryotic cell. The basic structural unit of chromatin is the nucleosome [1], which consists of 146 base pairs of double-stranded DNA wrapped around an octameric histone core complex [2]. The core complex is composed of two copies of each of the histone proteins H2A, H2B, H3, and H4, organized as a central (H3-H4)_2 _tetramer flanked by two H2A-H2B dimers [3]. Despite low sequence similarity, all core histone subunits share a common fold; they are composed of three helices separated by two short strap loops and assemble into heterodimers by interleaving the helices into the 'handshake motif' and juxtaposing the strap loops into short parallel β-bridges [3]. This fold may have arisen through the duplication of a primordial helix-strand-helix motif [4,5], consistent with the hypothesis that folded proteins arose by the combination of subdomain-sized peptides, the so-called antecedent domain segments [6-8].

Archaea also wrap their DNA into nucleosome-like structures [9]; their constituent histone subunits assemble into tetramers, which may reflect an ancestral form of the central part of the eukaryotic nucleosome octamer, the (H3-H4)_2 _tetramer [10]. Archaeal histone subunits are occasionally duplicated on a single polypeptide chain [11], a form observed in eukaryotes only in the histone-like domain of the son of sevenless protein [12].

Bacteria also have nucleoid proteins with histone-like properties [13], but these belong to a different, unrelated fold. However, a homolog of archaeal single-chain histones was recently reported from the bacterium *Aquifex aeolicus *(1R4V) [14]. Further homologs appear in the genomes of a few, phylogenetically diverse bacteria. It thus seems likely that the histone fold originated in the common ancestor of eukaryotes and archaea and spread into some bacteria through lateral gene transfer.

In an all-against-all application of HHsearch [15] to the SCOP database (JS, unpublished results) we found an evolutionary relationship between histone proteins and the helical part of the extended AAA+ ATPase domain, the C-domain [16,17]. Based on this finding, we used sequence and structure comparisons to reconstruct in detail the evolutionary events that may have shaped the histone fold. Our results point to a common origin not only with the C-domain but also with the N-terminal substrate recognition domain of Clp/Hsp100 proteins [18]. The conserved element is a helix-strand-helix motif, which we propose gave rise divergently to these three different folds and thus represents an antecedent domain segment.

## Results

Homology between proteins is typically inferred from similarities in sequence and structure. Sequence similarity is the primary criterion for deducing a common origin, but for distant evolutionary events, sequences may have diverged beyond our ability to detect their relatedness. Structures diverge much more slowly and their similarity is therefore often used to identify such distant events. However, similar structures may have arisen convergently from different origins and their similarity thus frequently does not provide conclusive evidence of common ancestry. In this study we applied a new, highly sensitive method for sequence comparison based on profile Hidden Markov Models (HMMs) to identify distant homologs of histones on the basis of sequence similarity alone. Subsequently, we validated our findings through structure comparisons.

### HMM-HMM comparisons

**Figure 1:**
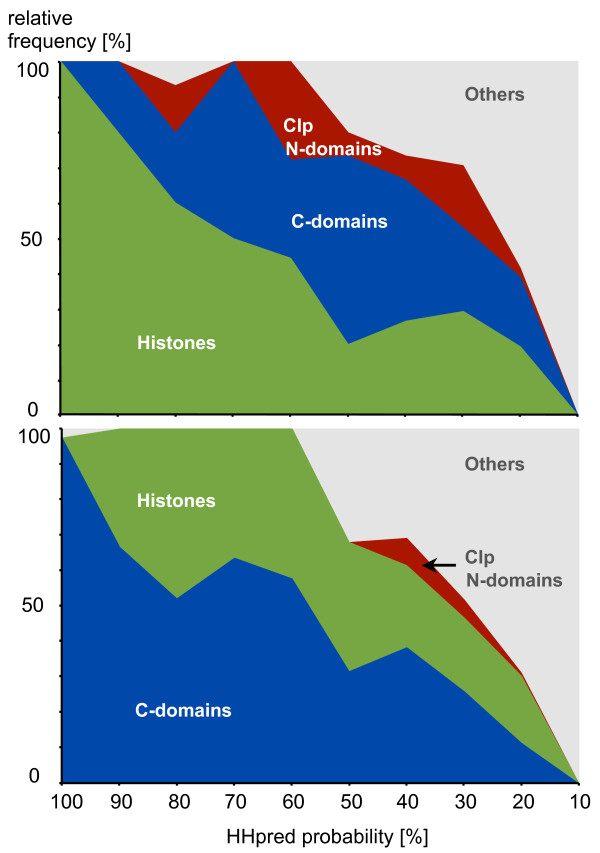
**Results of HHpred searches of the SCOP25 database with histone sequences and C-domains**. The relative frequencies of SCOP families encountered in the searches are plotted against the HHpred probabilities as described in the Methods (searches with histones – top panel; searches with C-domains – bottom panel). Histones (SCOP a.22) are colored in green, C-domains (SCOP c.37.1.20; also includes the misclassified a.49.1.1) in blue, Clp N-domains (SCOP a.174) in red and others in gray.

We used HHpred [15,19], a sensitive HMM-to-HMM comparison method, to detect homologs of the histone fold by searching the SCOP25 database [20] with sequences from the three protein families with this fold: archaeal histones, nucleosome core histones and TBP-associated factors. As expected, these identified each other as their best matches with high statistical significance (Fig. 1). Remarkably, their subsequent matches were consistently to the helical part of the extended ATPase domains found in AAA+ proteins (the C-domain) [16]. Good matches to a third protein family, the N-terminal domain of Clp/Hsp100 proteins (Clp N-domain), were frequently obtained [18]. Reciprocal searches with a set of C-domain sequences confirmed the similarity of these protein families (Fig. 1).

We found two high-scoring matches with other folds. These are an alanyl tRNA synthetase (1RIQ, a.203.1.1, identified by the histone entry 1JFI), and the zeta subunit of a plasmid maintenance system (1GVN, c.37.1.21, identified by two C-domains: 1LV7 and 1R7R). Subsequent analysis could not confirm these matches as homologs.

### Analysis of sequence and structure conservation

**Figure 2:**
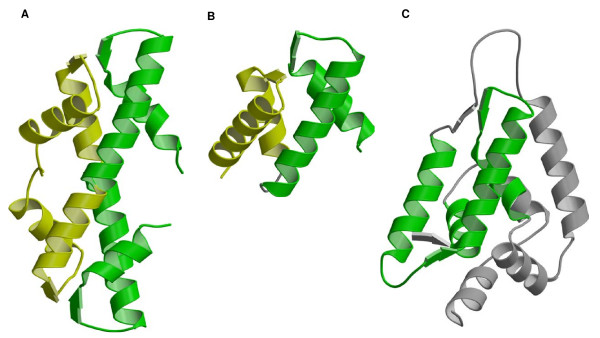
**The structure of histones, C-domains and Clp N-domains**. (A) The histone of *Methanothermus fervidus *(1B67); the N-terminal helix-strand-helix motif in each subunit is colored yellow and the C-terminal motif green. (B) The C-domain of the helicase RuvB (1IN4); the motifs are colored as in the histone subunits. (C) Clp N-domain of ClpA (1K6K); the two helix-strand-helix motifs are colored green.

The surprising aspect of these findings is that histones, C-domains and Clp N-domains belong to three different folds (Fig. 2A–C). Histones are dimeric, interleaved helical bundles, as described in the Background section. C-domains are four-helix bundles composed of two consecutive helix-strand-helix motifs [17]. Clp N-domains, finally, are multihelical domains formed by the repetition of a 4-helical motif [21]. Although these three protein families have different topologies, they all incorporate two copies of the helix-strand-helix motif, which engages in the formation of a short parallel β-bridge. In the histone dimer, the β-bridge is formed by the association of one helix-strand-helix motif from each monomer, in the C-domain by the association of the two motifs consecutive in the polypeptide chain, and in the Clp N-domains by the association of each motif with an N-terminal strand of the symmetry-related motif.

**Figure 3:**
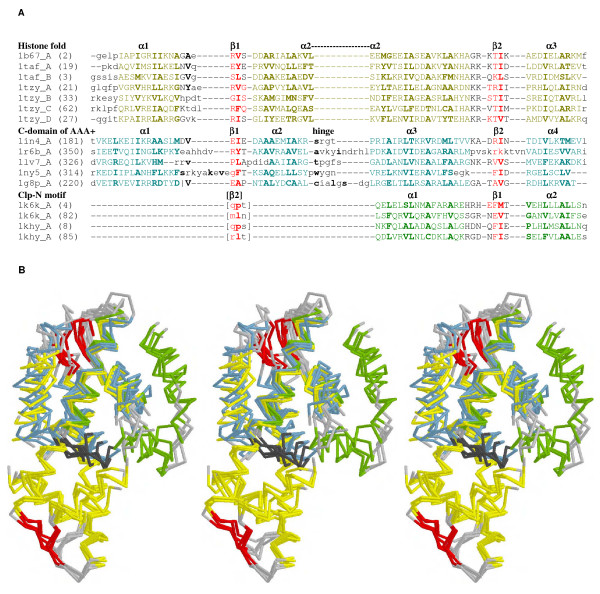
**Sequence and structure comparisons of histones, C-domains and Clp N-domains**. (A) Multiple sequence alignment of representative members of each fold. Residues in helices are colored yellow in histones, blue in C-domains, and green in Clp N-domains; residues in β-bridges are colored red. Structurally equivalent residues are shown in capital letters and residues forming the hydrophobic core are shown bold. The sequences are labeled by their PDB codes; the numbers in brackets refer to the residue number for the first residue in the alignment. (B) Global superposition of the proteins listed in panel A. The superposition was made using the archaeal histone HMfA (1B67) as a reference structure. Quantitative information on the results of the superposition is provided in Table 1. The coloring is as in panel A; in addition, the hinge region of C-domains is highlighted in black.

The similarities detected by HMM-to-HMM comparison are limited to these helix-strand-helix motifs. Histones and C-domains both contain two consecutive copies of the motif and can be aligned over essentially their entire length (Fig. 3A). Clp N-domains contain two motifs decorated by two helices and each motif has its best matches to the C-terminal motif of histones and C-domains (Fig. 3A). The sequence alignment shows extensive similarity in the hydrophobic patterns of the three folds, but no highly conserved residues other than two Alanines in the core of the second helix-strand-helix motif, which allow for close packing interactions at the crossover point between the helices.

A structural comparison of the three folds shows that C-domains can be superimposed onto one half of the histone fold with root-mean-square deviations (rmsd) of around 1.5Å (Table I). The main difference between the two folds lies in the fact that the two helix-strand-helix motifs of C-domains are connected by a hinge region, while they are continuous in histones, requiring dimerization to form the hydrophobic core (Fig. 3B). The similarity between histones and Clp N-domains is also in the range of 1.5Å rmsd, but extends only over the C-terminal helix-strand-helix motif of histones.

## Discussion

### Domain swapping as mechanism for protein evolution

The results presented here suggest an evolutionary link between histones and the C-domains of AAA+ proteins, despite differences in their topology. We propose 3D domain swapping as the mechanism that accounts for their structural differences. 3D domain swapping is a process by which two or more identical proteins exchange a domain to form interlocked oligomers [22], in which all of the packing interactions that stabilize the monomer are present. The swapped portions can range from a single secondary structure element to an entire domain. In the simplest case the native fold, normally constituted by a single 'closed' monomer, is reconstituted by two so-called 'open' monomers. This reciprocal swap leads to a homodimer, whereas the runaway domain swap, in which swapping propagates along an axis in an open-ended manner, has been proposed to contribute to amyloid fibril formation [23-25].

Up to now, about 40 proteins have been shown to be able to undergo 3D domain swapping [26], and several studies indicate a physiological role of this mechanism in allostery and signal transduction [27-29]. A precondition is the presence of a flexible loop or hinge, about which the swapped elements can rotate in order to form a pair of 'open' monomers. The primary intervention by which 3D domain swaps have been engineered into monomeric proteins is through the shortening of the hinge, thus preventing the packing of part of the protein into its native location and forcing a swap, such as in domain 1 of lymphocyte antigen CD2 [30], staphylococcal nuclease [31], single-chain Fv fragments [32,33], in a 3-helix bundle designed by Ogihara *et al*. [34].

**Figure 4:**
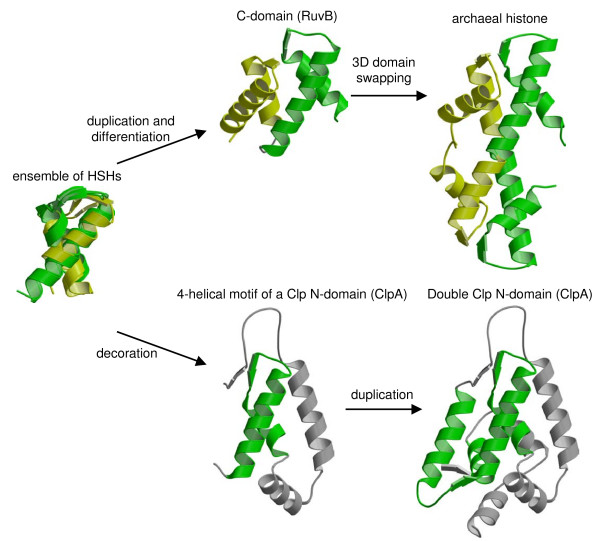
**Evolutionary scenario for the origin of three folds from an ancestral helix-strand-helix motif**. The coloring and representative proteins are as in Fig. 2. The superimposed ensemble of helix-strand-helix motifs consists of motifs from the following proteins: yellow (1IN4: residues 181–212; 1B67: 4–33), green (1IN4: 216–251; 1B67: 134–166; 1K6K: 82–115).

Our results suggest that such a shortening of the hinge region, which connects the two helix-strand-helix motifs of the AAA+ C-domain, led to a 3D domain swap. The event caused head-to-tail dimerization of monomers, which thereby recovered the lost interactions between the two helix-strand-helix motifs, and resulted in the emergence of the histone fold (Fig. 4). Following the proposal that domain swapping might contribute to protein evolution [22,35], we present here the first concrete example.

### A primordial helix-strand-helix motif

The helix-strand-helix motif, which is at the core of the similarity between histones and C-domains, is also found in Clp N-domains, which assume yet a third fold. Here, the motif is decorated with two C-terminal helices, and two copies of this extended, 4-helical motif are fused in antiparallel orientation. Thus, three different folds appear to have been built from a common helix-strand-helix motif. One theory for the origin of folded proteins proposes that they arose by fusion and recombination from an ancestral set of peptides, which emerged in the context of RNA-dependent replication and catalysis (the 'RNA world') [6-8]. The helix-strand-helix motif would be such an ancestral peptide, which gave rise divergently to the Clp N-domain and the AAA+ C-domain through two independent events of duplication and fusion (Fig. 4). The C-domain then evolved into the histone fold by 3D domain swapping. This scenario extends a previous hypothesis on the origin of eukaryotic core histones, which proposed that they evolved from the duplication of a single helix-strand-helix motif [4,5].

In this study we have deduced homology based on similarities in sequence and structure. We are aware that homology of proteins is an assumption inferred from heuristics, of which sequence similarity is generally accepted as the best indicator. Structural similarity alone, especially of small fragments, does not necessarily imply evolutionary divergence, since it may result from general biophysical constraints. Indeed, we find a number of α-helical hairpins in the PDB with a high degree of structural similarity to the helix-strand-helix motif (rmsds of less than 1.5Å); some examples include hairpins from fumerate reductase (1QLA_A, residues 65–94) and tetracycline repressor-like protein (1T33_A, residues 144–173). However, none of them show detectable sequence similarity to each other or to the proteins in our study. This shows that the constraints of structure on sequence variability are not sufficient to explain the observed sequence similarity between histones, C-domains, and Clp N-domains.

### Functional implications

**Figure 5:**
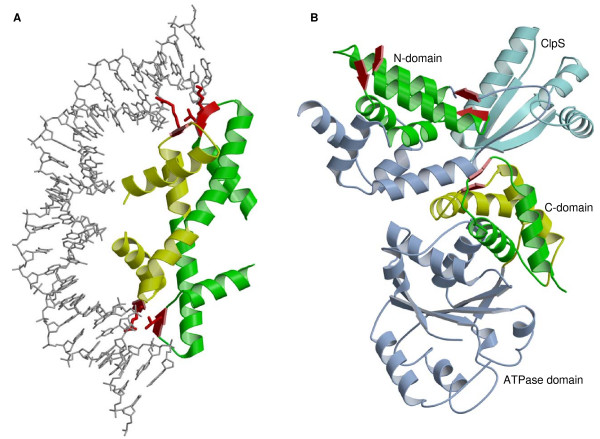
**Involvement of the β-bridge in macromolecular interactions**. The coloring of helix-strand-helix motifs is as in Fig. 2, except the β-bridges are colored red. (A) The histone H3-H4 complex bound to DNA (1S32); residues of the β-bridges engaged in interactions with the phosphate backbone are shown in stick representation. (B) ClpS in complex with ClpA (1LZW, 1R6B); the ATPase domain is in light blue and ClpS in cyan.

An interesting structural feature common to all three folds is the presence of one or two short, parallel β-bridges formed by the strands of the helix-strand-helix motifs. In histones, these β-bridges provide the main site of interaction with the phosphate backbone of DNA (Fig. 5). In Clp N-domains, one of the two β-bridges binds the adaptor molecule ClpS [18,21] (Fig. 5). Although the binding sites of the AAA+ C-domains have not been characterized yet, it thus seems attractive to propose that here also the single β-bridge formed in this domain represents the main binding site. C-domains play an important role in sensing the nucleotide bound by the AAA+ proteins [36-38] and are located close to the substrate-binding N-domains (Fig. 5), projecting radially at the circumference of the hexameric ring complex. We note in this context that C-domains are frequently rich in positively charged residues and that in the Lon protease, the C-domain has been implicated in interactions with DNA [39]. We propose that the helix-strand-helix motif served as a scaffold for the formation of parallel β-bridges. Ancestrally, these bridges bound proteins, but in a few C-domains they also acquired the ability to bind DNA, eventually leading to histones as proteins that only bind DNA at these sites.

## Conclusion

We have retraced the evolutionary events which may have shaped the histone fold and have found connections to two other folds; the N-terminal substrate recognition domain of Clp/Hsp100 proteins and the helical part of the extended AAA+ ATPase domain. These 3 folds contain a homologous helix-strand-helix motif, despite the differences in the topology, leading us to propose a scenario for the origin of these folds from a common ancestral helix-strand-helix motif through events of duplication, fusion and 3D domain swapping. The short functional parallel β-bridges formed by the strands of the helix-strand-helix motifs seem to be the evolutionary driving force for the conservation of this motif. Our findings provide additional support for our previously proposed hypothesis that the diversity of today's folds might have arisen from an ancestral set of peptides.

## Methods

We obtained histone and Clp N-domain sequences from the ASTRAL compendium [40] as defined by the SCOP (version 1.71) [20] folds a.22 and a.174, respectively, and reduced the set to less than 25% pairwise identity at 90% length coverage using BLASTCLUST [41]. C-domains are not characterized as a separate fold in SCOP; we extracted their sequences from the 'extended AAA-ATPase' family (c.37.1.20) of the SCOP database by a procedure described by Ammelburg et al. [17] and also reduced this set to less than 25% pairwise identity.

We used these sequences to search the SCOP25 database for homologs with HHpred [15,19], at default parameters and a probability cutoff of 10%. The SCOP25 database is a version of SCOP filtered for a maximum of 25% pairwise sequence identity. For each group, we pooled all search results and tabulated the frequencies at which various SCOP families appeared at each probability, binned at 10% intervals.

The histone, C-domain and Clp N-domain structures were superimposed interactively in Swiss-PDB viewer [42]. We chose the archaeal histone HmfA (1B67) as the reference structure, as it made the highest number of connections both in sequence and structure searches. Quantitative information for the superimposition is listed in Table 1. The alignment in Fig. 3A reflects the structural superposition. The complex shown in Fig. 5B, consisting of ClpS, N-domain and the first AAA+ domain of ClpA, was generated by superimposing the N-domains of the structures 1R6B (N-domain and the AAA+ domains) and 1LZW (N-domain in complex with ClpS) from *E. coli*.

## Competing interests

The author(s) declare that they have no competing interests.

## Authors' contributions

VA and MA contributed equally to this work. JS discovered the similarity between histones and C-domains. VA, MA and AL conceived this study and VA and MA executed the analysis. VA, MA and AL wrote the paper. All authors read and approved the final manuscript.

## Figures and Tables

**Table 1:** Data for the superposition in Fig. 3

**PDB-ID**	**Name**	**SOURCE SPECIES**	**FOLD**	**SUBGROUP**	**NO. OF ALIGNED RESIDUES**	**RMSD TO HMFA [Å]**
1B67 A+B	HmfA	*Methanothermus fervidus*	Histone	Archaeal	124/124	0.00
1TAF A+B	TAFII42/62	*Drosophila melanogaster*	Histone	TBP-associated factors	124/132	1.19
1TZY A+B	H2A/H2B	*Gallus gallus*	Histone	Nucleosome core	118/129	1.33
1TZY C+D	H3/H4	*Gallus gallus*	Histone	Nucleosome core	114/134	1.10
1IN4 A	RuvB	*Thermotoga maritima*	C-domain	AAA+: helicases	46/71	1.38
1R6B A	ClpA-D1	*Escherichia coli*	C-domain	AAA+: Clp-D1	52/86	1.70
1LV7 A	FtsH	*Escherichia coli*	C-domain	AAA+: AAA	45/71	1.21
1NY5 A	NtrC	*Aquifex aeolicus*	C-domain	AAA+: σ 54	52/72	1.57
1G8P A	Mg chelatase	*Rhodobacter capsulatus*	C-domain	AAA+	a) 12/16(α 1) b) 47/54(α 2–4)	a) 0.59 b) 1.38
1K6K A	ClpA-N (1^st ^half)	*Escherichia coli*	Clp N-domain	ClpA	33/78	1.54
1K6K A	ClpA-N (2^nd ^half)	*Escherichia coli*	Clp N-domain	ClpA	33/62	1.40
1KHY A	ClpB-N (1^st ^half)	*Escherichia coli*	Clp N-domain	ClpB	33/80	1.13
1KHY A	ClpB-N (2^nd ^half)	*Escherichia coli*	Clp N-domain	ClpB	33/58	0.96
